# Comparison of Clinical and Radiological Outcomes between Calibratable Patient-Specific Instrumentation and Conventional Operation for Medial Open-Wedge High Tibial Osteotomy: A Randomized Controlled Trial

**DOI:** 10.1155/2022/1378042

**Published:** 2022-11-23

**Authors:** Fawei Gao, Xucheng Yang, Chenggong Wang, Shilong Su, Jun Qi, Zhigang Li, Juehao Chen, Da Zhong

**Affiliations:** ^1^Department of Orthopaedics, Xiangya Hospital, Central South University, Changsha 410008, China; ^2^Department of Orthopaedics, Dali Bai Autonomous Prefecture People's Hospital, Dali 671000, China; ^3^Department of Orthopaedics, Peking University Third Hospital, Beijing 100191, China

## Abstract

**Background:**

High tibial osteotomy (HTO) is an effective surgery in treating medial compartment knee osteoarthritis (KOA) combined with varus deformity. An accurate orthopaedy is the key and challenge to the success of HTO. Therefore, we designed a calibratable patient-specific instrumentation (PSI) to assist surgery and evaluated its accuracy and clinical outcomes by comparing with conventional operation (CO).

**Materials and Methods:**

37 patients (39 knees) with medial compartment KOA were randomly divided into the PSI and CO groups and underwent medial open-wedge high tibial osteotomy (MOWHTO) from September 2020 to May 2021. The postoperative radiological outcomes were compared with the preoperative measurements or target values to evaluate the accuracy of correction in the two groups. The American Knee Society Score (AKSS), complication rate, number of intraoperative radiation exposures, blood loss volume, and operative duration were analysed to evaluate the clinical outcomes in the two groups.

**Results:**

The designed target values were better achieved in the PSI group than in the CO group. The mean absolute difference between the postoperative measurements and preoperative targets was significantly lower in the PSI group than in the CO group (weight-bearing line (WBL) ratio, 1.97 ± 1.83% vs.5.42 ± 4.41%, *P* = 0.002; hip-knee-ankle (HKA) angle, 1.12 ± 0.86° vs. 2.27 ± 1.97°, *P* = 0.018). The operative duration was significantly shorter (*P* = 0.014), and the number of radiation exposures (*P* < 0.001) and volume of intraoperative blood loss (*P* = 0.003) were significantly lower in the PSI group than in the CO group. The clinical AKSS score at 3 and 6 months postoperatively and the functional AKSS score at 3 months postoperatively were significantly higher in the PSI group than in the CO group (*P* = 0.042, 0.040, and 0.034, respectively).

**Conclusion:**

For patients with medial compartment KOA, calibratable PSI can assist the surgeon in MOWHTO with superior accuracy and clinical efficacy. This study was conducted under Randomized Controlled Trial Details (RCT) with Registry Number ChiCTR2000038619.

## 1. Introduction

With the standardization of high tibial osteotomy (HTO) surgical techniques and the continuous development of plate fixators, surgical treatment with HTO for young active patients with medial knee osteoarthritis (KOA) combined with tibial varus deformity has become a very appropriate option for knee preservation [1]. Favourable long-term outcomes have been reported by numerous studies, and medial open-wedge high tibial osteotomy (MOWHTO) has been more commonly applied among the different surgical approaches [2, 3].

The correction angle is determined in conventional HTO by using full-length weight-bearing radiographs of the lower limbs, which are then evaluated intraoperatively by reiterative radiation exposures in non-weight-bearing positions and the size of the open wedge to ensure satisfactory alignment of the lower limb. The disadvantages of this method include intraoperative assessment with only two-dimensional (2D) coronal correction, the susceptibility of real-time monitoring of the weight-bearing line (WBL) ratio to being affected by limb rotation, and the greater number of intraoperative radiation exposures, which result in inadequate correction accuracy [4–6].

With the rapid advancement of three-dimensional (3D) printing technology, various types of 3D-printed patient-specific instrumentation (PSI) have been used in HTO, allowing easier and more accurate osteotomy, and reducing the operative duration, learning curve for surgeons, and number of intraoperative radiation exposures [6–10]. However, some of these types required extensive soft tissue dissection due to the large volume of the PSI. Additionally, there was the problem that reducing the PSI volume would lead to a reduction in the fit with the bone surface, which could affect the accuracy of the PSI. Therefore, further improvements could be achieved in terms of reducing trauma and improving accuracy. Additionally, the current PSI mainly consists of a guide to assist with osteotomy, which lacks calibration of the correction angle and WBL ratio.

In this study, calibratable 3D-printed PSI consisting of a proximal osteotomy guide and a distal calibratable guide is described. The aims of this design were to reduce the PSI volume and easily obtain a reliable bony surface compatible with the fitting of the PSI while reducing soft tissue damage and facilitating the accurate and rapid implementation of HTO. The purpose of this research was to evaluate the accuracy and clinical outcomes of calibratable 3D-printed PSI by comparing it with conventional operation (CO).

## 2. Materials and Methods

### 2.1. Patient Selection and Randomized Grouping

**Figure 1:**
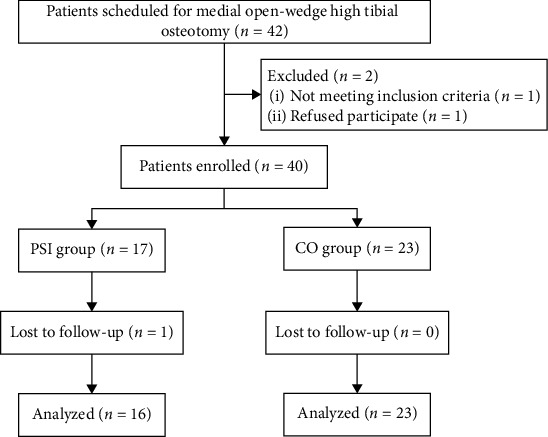
Flow diagram of patient selection and exclusion.

In this prospective, randomized controlled trial (RCT), when each patient was enrolled, the investigator assigned the patient to either the PSI or CO group based on pre-established grouping rules and a random number generated by computer software (Excel, Microsoft Corporation, Redmond, USA). Finally, 37 patients (39 knees) with medial KOA were ultimately enrolled from September 2020 to May 2021, including 16 patients (16 knees) in the PSI group and 21 patients (23 knees) in the CO group (Figure 1, Table 1). The results were only provided to the chief surgeon, who did not participate in the postoperative follow-up data collection or statistical analysis. Follow-up data collectors and statistical analysts were blinded to the group allocations, and patients were informed of the group to which they belonged at the end of the RCT. This study was approved by the authors' institutional review board (No.202008253) and registered at the Chinese Clinical Trial Registry (ChiCTR2000038619). Informed consent was obtained from all patients included in the study.

The inclusion criteria of this study were as follows: (1) age ≤65 years for females and ≤68 years for males; (2) body mass index (BMI) < 30 kg/m^2^ and medial KOA with pain located at the medial compartment of the knee joint that was not responsive to conservative treatment; (3) varus deformity originating from the proximal tibia demonstrated on imaging and a medial proximal tibial angle (MPTA) < 85°; (4) range of knee flexion and extension >90° and knee flexion contracture deformity <20°. The exclusion criteria of this study were as follows: (1) gouty arthritis, rheumatoid arthritis, or haemophilic arthritis with no osteoarthritis; (2) poor knee stability, poor muscle strength in the lower limbs, or osteoporosis; (3) other comorbidities or inability to tolerate surgery.

### 2.2. Preoperative Planning

**Figure 2:**
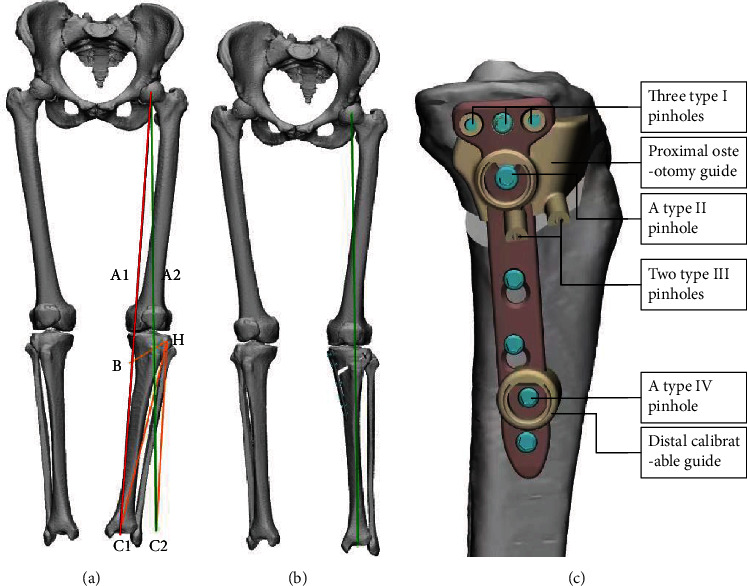
Preoperative planning. (a) Preoperative design. Line A1: preoperative WBL ratio. Line A2: target WBL ratio. The WBL ratio in this case was 62.5%. Point H: lateral hinge point. Point B: cutting point of the first osteotomy plane. ∠C1HC2: target correction angle. (b) Simulation of the corrective effect. (c) Design of PSI. (WBL: weight-bearing line.).

Preoperative full-length anteroposterior weight-bearing radiographs of the bilateral lower limbs, anteroposterior, and lateral radiographs of the affected knee were obtained in both groups. In the PSI group, computed tomography (CT) of the lower limbs, including both hips, knees, and ankles, was performed with a slice thickness of 0.8 mm to reconstruct a 3D model with Mimics 25.0 software (Materialize, Leuven, Belgium). The preoperative WBL ratio (line A1) was marked in the reconstructed 3D model, and the target WBL ratio (line A2) was designed to pass through 55.0-62.5% of the tibial plateau from medial to lateral (Figure 2(a)), and the lateral hinge (H) was designed at the upper 1/3 of the fibular head (approximately 1.5 cm below the lateral tibial plateau and 1 cm away from the proximal lateral tibial cortex) (Figure 2(a)); then, the two lines were connected from the lateral hinge (H) to the centre of the preoperative and corrected ankle joints (C1 and C2), respectively (Figure 2(a)). The angle formed by these two lines was the target correction angle (Figure 2(a)) [11, 12].

Then, the biplanar osteotomy was designed using the reconstructed 3D model. The cutting point (B) of the first osteotomy plane (horizontal cut) was located above the pes anserinus and approximately 4 cm below the medial tibial plateau, with the horizontal cut extending from the cutting point to the lateral hinge (Figure 2(a)). The second osteotomy plane was located behind the tibial tuberosity and approximately parallel to the anterior cortical edge of the tibia. The angle between the horizontal osteotomy line and the sagittal osteotomy line was approximately 110° [13]. Finally, the corrective effect was simulated, and osteotomy correction was simulated by the computer according to the preoperative design to check the position of the corrected WBL ratio (Figure 2(b)). The target values of the design were recorded, including the WBL ratio, hip-knee-ankle (HKA) angle, MPTA, and correction angle.

**Figure 3:**
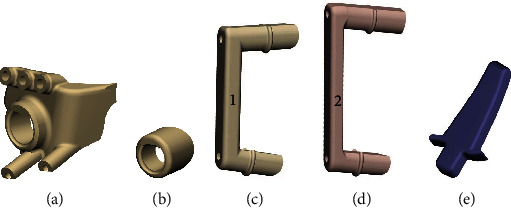
Components of PSI. (a) Proximal osteotomy guide. (b) Distal calibration guide. (c) Connecting rod. (d) Calibratable rod. (e) Positioning wedge block.

The installation position of the plate fixator was preset after the osteotomy planning was completed, and the orthopaedic effect was simulated. First, the proximal osteotomy guide was designed, in which three type I pinholes were designed to correspond with holes A-C of the plate fixator and fix the PSI during the osteotomy. A type II pinhole was designed to correspond with hole D of the plate fixator, and two type III pinholes were designed on the distal side parallel to the osteotomy surface (Figure 2(c), Figure 3(a)). Second, the distal calibratable guide was designed, in which a type IV fixation pinhole was designed to correspond with the 3rd hole at the distal side of the plate fixator (Figure 2(c), Figure 3(b)). Finally, we designed a connecting rod, calibratable rod, and positioning wedge block (Figures 3(c)–3(e)). These designed components of the PSI were sent to a 3D printer (FLIGHT HT403P, Hunan Farsoon High-Technology Co., Ltd.) in STL format for printing of the PSI using a high-temperature and high-pressure-resistant nylon polymer.

### 2.3. PSI Structure and Function

**Figure 4:**
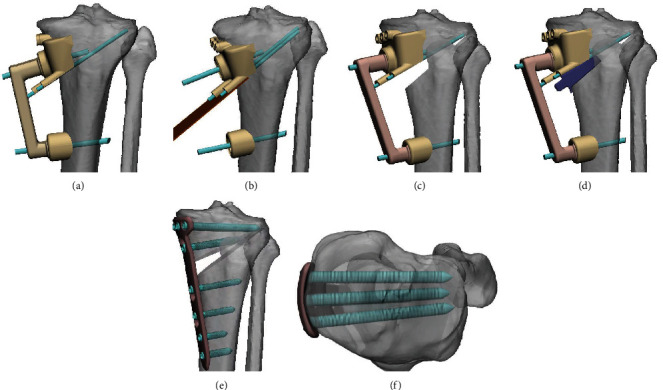
Simulation of the surgical procedures. (a) Installation of the PSI before the osteotomy. (b) Biplanar osteotomy with the aid of the osteotomy guide. (c, d) Calibration after the osteotomy. (e, f) Installation of the plate fixator under the guidance of the positioning wedge block. (PSI: patient-specific instrumentation. PTSA: posterior tibial slope angle.).

This calibratable PSI mainly consists of a proximal osteotomy guide, distal calibration guide, connecting rod, calibratable rod, and positioning wedge block (Figures 3(a)–3(e)). The proximal osteotomy guide and distal calibration guide were located at each end of the connecting rod and fixed to the tibia by Kirschner wires, which were used to assist in determining the position, angle, and depth of the osteotomy (Figures 4(a)–4(b)). After completing the osteotomy and bracing, the calibratable rod was connected between the proximal osteotomy guide and distal calibration guide to check the correction angle (Figure 4(c)). Then, the positioning wedge block was inserted into the open wedge to assist in checking the correction angle, supporting the gap, and installing the plate fixator (Figure 4(d)–4(f)).

### 2.4. Preoperative Planning in the CO Group

In the CO group, patients' preoperative full-length anteroposterior weight-bearing radiographs of the bilateral lower limbs were imported into computerized osteotomy simulation software (Osteo Master) to plan the osteotomy, and the range of the target WBL ratio, the lateral hinge, and the cutting point was designed to be the same as those in the PSI group. Meanwhile, the WBL ratio, HKA angle, MPTA, correction angle, and posterior tibial slope angle (PTSA) were recorded.

### 2.5. Surgical Technique

**Figure 5:**
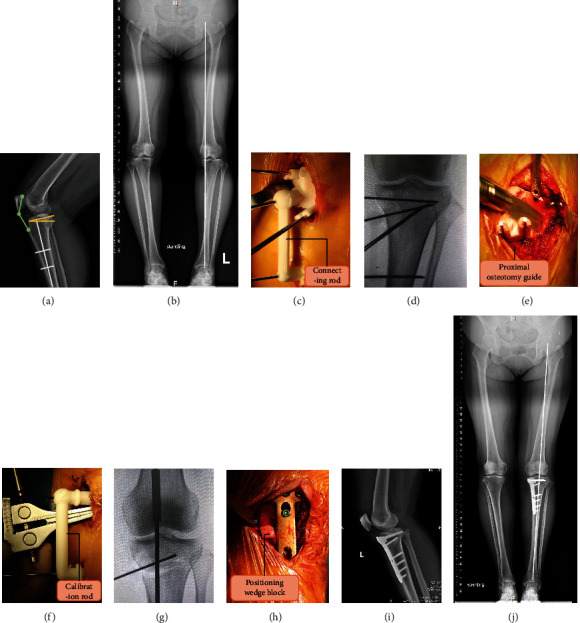
Surgical technique of PSI. (a, b) Preoperative imaging. The Insall–Salvati index is indicated by the green lines: ratio of the length from the inferior pole of the patella to the superior border of the tibial tuberosity and the longest diagonal length of the patella. The PTSA is indicated by the yellow lines. (c) Installation and fixation of the proximal osteotomy guide. (d) X-ray fluoroscopy to confirm the position of the guide. (e) Biplanar osteotomy according to the orientation of the osteotomy guide and Kirschner wires. (f) Calibration of the open wedge with the aid of calibratable rod. (g) Intraoperative radiography to assist in confirming the alignment of lower limb. (h) Installation of the plate fixator under the guidance of the positioning wedge block. (i, j) Postoperative imaging. (PSI: patient-specific instrumentation. PTSA: posterior tibial slope angle.).

All procedures were performed by two senior surgeons. The patients were placed in a supine position; sterilization and surgical draping were performed after satisfactory nerve block anaesthesia was achieved. After opening the tourniquet, a segmented longitudinal incision was made at the proximal medial tibia (approximately 5 ~ 7 cm proximally and 1 cm distally). The skin and subcutaneous tissues were separated sequentially. In the PSI group, the proximal osteotomy guide was installed, and its proper positioning was confirmed by both its fit to the bone surface and the direction of the Kirschner wires in intraoperative radiographs; then, the position of the distal calibration guide was confirmed and fixed using the connecting rod (Figure 4(a), Figures 5(c)–5(d)). Subsequently, biplanar osteotomy was performed in the orientation of the proximal osteotomy guide according to the preoperatively designed targets (Figure 4(b), Figure 5(e)). Then, stacked bone cutters were inserted sequentially, and the calibratable rod was connected when the preset angle or wedge height reached the expected value. The wedge opening was stopped when the calibratable rod connected the proximal osteotomy guide and the distal calibration guide (Figure 4(c), Figure 5(f)). Finally, with the assistance of a positioning wedge block, the calibration was checked again, and a radiograph was obtained to assess whether the alignment was satisfactory (Figure 5(g)). The plate fixator was implanted after satisfactory correction was achieved and fixed with screws (Figures 4(e)–4(f), Figure 5(h)). Intraoperatively, bone grafting was performed if the open wedge was more than 1 cm, and a wound drain was implanted if the bleeding was heavy.

In the CO group, the general surgical preparation procedures and the cutting point were approximately the same as those in the PSI group. The direction of the intraoperative osteotomy was determined by repeated radiography, the depth of the osteotomy was evaluated by the surgeon based on experience, the height and angle of the open wedge was adjusted according to the preoperative plan and intraoperative alignment, and the placement of the plate fixator was performed according to the experience of the surgeon. The standards for bone grafting and drainage were similar to those in the PSI group. The operative duration, number of radiation exposures, intraoperative blood loss volume, and use of bone grafting were all recorded during the surgery.

### 2.6. Postoperative Management and Outcome Evaluation

In both groups, patients were treated postoperatively with general therapy, including anti-inflammatories, analgesics, and detumescence. On the first day after surgery, patients started to perform quadriceps contraction training, active and passive knee flexion and extension activities, and standing on the ground with the assistance of double crutches. The limb on the operative side was allowed contact with the ground, with no obvious pain as a principle. Patients walked with a single crutch at 4 weeks postoperatively and walked without crutches 6-8 weeks after surgery according to the healing of the osteotomy on knee X-ray examination and then gradually returned to normal life [14, 15].

In both groups, anteroposterior and lateral radiographs of the affected knee and full-length anteroposterior weight-bearing radiographs of bilateral lower limbs were obtained 1 to 3 days after surgery. The WBL ratio, HKA angle, MPTA, correction angle, PTSA, and Insall–Salvati index were measured and compared with the preoperative measurements or designed target values to evaluate the accuracy of the surgery in both groups. The patients' knee function was evaluated with the American Knee Society Score (AKSS) preoperatively, at 3 and 6 months postoperatively, and at the last follow-up. The operative duration, number of radiation exposures, intraoperative blood loss volume, use of bone grafting, and complication rate were compared between the two groups.

### 2.7. Statistical Analysis

Statistical software SPSS 25.0 (SPSS, USA) was used for data analysis. Continuous data were tested for normality, and normally distributed were expressed as the mean and standard deviation. Paired *t* tests were used for comparison of the PTSA and Insall–Salvati index before and after surgery, as well as for comparison of the preoperatively designed targets and corresponding postoperative values in each group, which included the WBL ratio, HKA angle, MPTA, and correction angle. Two-sample t tests were used to compare continuous data between the two groups. Categorical data, including sex and surgery side, were compared by Fisher's exact test using a 2 × 2 table. The AKSS score at each time point before and after surgery was compared between the two groups using two-sample *t* tests. Statistical significance was assumed at *P* < 0.05.

## 3. Results

### 3.1. Radiological Outcomes

**Figure 6:**
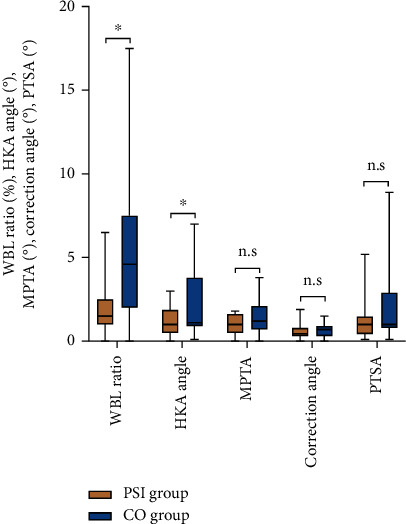
Absolute difference between postoperative measurements and preoperatively designed targets (WBL ratio, HKA angle, MPTA, and Correction angle)/preoperative values (PTSA). ^∗^*P* < 0.05; n.s.: no significance. (WBL: weight-bearing line. HKA: hip-knee-ankle. MPTA: medial proximal tibial angle. PTSA: posterior tibial slope angle).

The WBL ratio was corrected from 25.33 ± 5.66% preoperatively to 58.94 ± 3.35% postoperatively in the PSI group and from 24.49 ± 3.21% to 56.67 ± 8.03% in the CO group, with no significant difference in the postoperative measurements between the two groups (*P* = 0.234). The difference between the postoperative measurements and preoperative target values was not statistically significant in the PSI group (*P* = 0.063) but was statistically significant in the CO group (*P* = 0.017). The absolute value of the difference between the postoperative and preoperative target WBL ratio in the PSI group was lower than that in the CO group (*P* = 0.002) (Table 2, Figure 6).

The HKA was corrected from 173.00 ± 1.95° preoperatively to 182.86 ± 1.20° postoperatively in the PSI group and from 173.83 ± 2.87° to 181.73 ± 2.69° in the CO group, with no significant difference in the postoperative measurements between the two groups (*P* = 0.085). Furthermore, although the postoperative measurements in the PSI and CO group did not meet the preoperatively designed targets (*P* = 0.038, 0.003, respectively), the absolute difference between the postoperative measurements and the preoperative target was smaller in the PSI group than in the CO group (*P* = 0.018) (Table 2, Figure 6).

Regarding the MPTA and correction angle, the differences in the postoperative measurements between the two groups were not statistically significant (*P* = 0.165, 0.868, respectively), and the preoperative target values were achieved in both groups (Table 2, Figure 6). For the PTSA, although there was no significant difference in the postoperative PTSA between the two groups (*P* = 0.866), the postoperative PTSA in the CO group was significantly greater than the preoperative measurements (*P* = 0.043), while that in the PSI group was comparable to the preoperative values (*P* = 0.969). Concerning the Insall–Salvati index, there were no significant differences between before and after surgery in the same group or between the two groups (*P* > 0.05) (Table 2, Figure 6).

### 3.2. Clinical Outcomes

Regarding the intraoperative parameters, the total operative duration (time from skin incision to wound closure) was shorter in the PSI group (109.38 ± 20.83 minutes) than in the CO group (131.65 ± 29.92 minutes) (*P* = 0.014), the number of intraoperative radiation exposures was lower in the PSI group (18.50 ± 4.80) than in the CO group (28.22 ± 4.28) (*P* < 0.001), and the volume of intraoperative blood loss was lower in the PSI group (50.63 ± 14.36 mL) than in the CO group (97.82 ± 67.35 mL) (*P* = 0.003). There was no significant difference in the intraoperative use of bone morphogenetic protein (BMP) or bone grafting between the two groups (*P* = 0.516). One patient in the PSI group suffered from incisional exudation two weeks after surgery, and two patients in the CO group suffered from a nondisplaced hinge fracture intraoperatively. No neural injuries or postoperative infections occurred in either group (Table 3).

**Figure 7:**
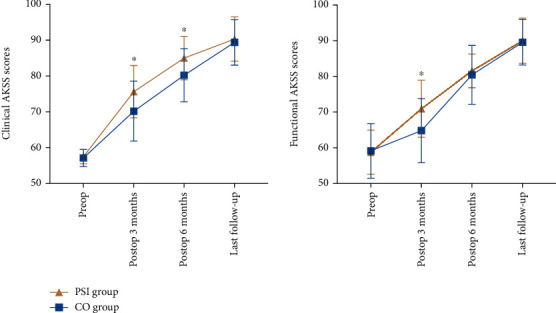
Patients' functional scores before and after surgery. ^∗^*P* < 0.05. (AKSS: American Knee Society Score. Preop: preoperative. Postop: postoperative).

The clinical AKSS score was higher in the PSI group than the CO group at 3 and 6 months postoperatively (75.63 ± 7.27 vs. 70.17 ± 8.37, *P* = 0.042 and 85.00 ± 6.06 vs. 80.22 ± 7.42, *P* = 0.040), and the functional AKSS was higher in the PSI group than in the CO group at 3 months postoperatively (70.93 ± 8.00 vs. 64.78 ± 8.98, *P* = 0.034). No statistically significant differences were observed in the AKSS score between the two groups of patients at other follow-up time points (*P* > 0.05) (Table 3 and Figure 7).

## 4. Discussion

HTO aims to restore the normal WBL ratio of the affected limb, reduce the stress on the medial compartment of the knee joint, and effectively relieve further damage to the cartilage and joint degeneration to relieve the symptoms of medial knee arthritis [12, 16, 17]. Accurate realignment of the WBL ratio is both the key to ensuring favourable long-term outcomes of HTO and currently the main challenge in HTO [17–19].

Conventional HTO is highly dependent on the surgeon's experience. Intraoperative assessment and adjustment of alignment and angles are performed with repeated X-ray fluoroscopy. Mechanical axis assessment is often affected by limb rotation and blockage of the visual field, and it is difficult to take into consideration of 3D correction; therefore, conventional HTO has shortcomings, such as limited correction accuracy, a larger number of radiation exposures, and a longer operative duration [4–6, 11, 20]. Inaccurate surgery may easily cause fracture of the tibial plateau or the hinge or a change in the PTSA, which may affect the efficacy of HTO [21].

In recent years, technologies such as computer navigation and the 3D printing of PSI have been applied in HTO. It was reported that the application of computer navigation-assisted HTO yielded satisfactory correction [22, 23]. A recent meta-analysis by Cerciello et al. [24] showed that the application of computer navigation-assisted HTO can reduce the occurrence of abnormal correction compared with conventional surgery, but the accuracy of correction was not significantly improved. In addition, computer navigation suffers from several disadvantages, including registration failure, software failure, a long learning period, and high cost [9]. The integration of HTO surgical planning with 3D-printed PSI has become a research hotspot in recent years, and positive corrective results have been reported [8, 11, 25]. A study by Fucentese et al. [26] demonstrated that using PSI to assist in HTO was beneficial for obtaining the desired correction of the HKA angle and maintaining a constant PTSA. A prospective cohort study by Jacquet et al. [10] indicated that the application of PSI-assisted HTO optimized the operative duration, reduced the need for intraoperative fluoroscopy, and shortened the learning curve while ensuring correction accuracy. However, some structures and function of current reported PSI are still to be improved. On the one hand, the use of some PSI is more traumatic because of the large volume of PSI, which requires more soft tissue stripping; additionally, the incompleteness of the extensive stripping in turn affects the adhesion of the PSI to the bony surface, thus influencing the accuracy of the correction. On the other hand, the current types of PSI are mostly designed to guide osteotomy, which involves insufficient calibration of the correction angle and alignment. Therefore, it is possible to improve PSI in terms of trauma, functional comprehensiveness, and correction accuracy.

The PSI used in this study was designed with a calibratable structure, including a proximal osteotomy guide and a distal calibration guide. First, the calibratable structure design reduced the volume of each part of the PSI while avoiding the need to separate critical soft tissue structures, such as the pes anserinus, thereby reducing soft tissue damage while obtaining a reliable fit. Second, the proximal osteotomy guide indicated the position, angle, and depth of the osteotomy, and the distal calibration guide further assisted in determining the angle of correction and alignment to improve the accuracy of correction. In addition, the type I, II, and IV pinholes of the proximal osteotomy guide and the distal calibratable guide corresponded with the screw holes of the plate fixator, and the positioning wedge block was used to maintain the correction angle and assist in placement of the plate fixator.

The commonly recommended WBL ratio in HTO is 62.5% through the lateral tibial plateau (Fujisawa point), but there is some debate about the ideal correction target to be achieved after HTO; 62.5% is not fully advocated as the only target value for every patient, and realignment of the WBL ratio to 55-60% might be considered a “safe target” [12]. The preoperative target WBL ratio adopted in this study was 55-62.5%, and the postoperative WBL ratio (58.94 ± 3.35%) differed from the preoperative target by an absolute value of 1.97 ± 1.83% in PSI group. However, this difference was not statistically significant and is not only comparable to the difference (2.3 ± 2.5%) in the WBL ratio obtained after the application of PSI-assisted HTO compared with the preoperative target reported by Kim et al. but also superior to that obtained in conventional surgical groups [11]. The radiological results obtained in this study indicate that an accurate correction angle could be obtained in the CO group using conventional surgical tools; the discrepancy in the target WBL ratio may originate from inaccuracy in the intraoperative osteotomy direction or depth or in the intraoperative fluoroscopy. In addition, the absolute difference in the HKA angle between preoperative target and postoperatively was lower in the PSI group than in the CO group, resulting in better biplanar correction in the PSI group than in the CO group without changing the PTSA. Moreover, the results of the study indicate that the use of PSI could shorten the overall operative duration, reduce the degree of surgical trauma, reduce the number of radiation exposures, and yield better early clinical results.

There are still some limitations to this study: (i) further investigation with a large sample size is required, as the main purpose of the study was to investigate the accuracy of calibratable 3D-printed PSI in MOWHTO; (ii) the follow-up time is short, and a longer follow-up period is needed to observe the intermediate and long-term efficacy.

## 5. Conclusion

The use of calibratable 3D-printed PSI in MOWHTO yields greater orthopaedic accuracy while reducing the difficulty of the operation, amount of soft tissue damage, repeated use of intraoperative fluoroscopy to adjust the WBL ratio, and number of osteotomies, thereby shortening the operative duration and demonstrating great practicality.

## Figures and Tables

**Table 1:** Demographic characteristics of the two groups.

	PSI group (*n* = 16)	CO group (*n* = 23)	*P* value
Age and years	60.81 ± 4.02	56.21 ± 9.4	0.074
Sex (male : female)	6 : 10	10 : 13	0.752
Side (right : left)	10 : 6	7 : 16	0.059
BMI (kg/m^2^)	24.72 ± 2.97	24.49 ± 3.21	0.823
Kellgren–Lawrence grade (I:II:III)	1 : 4 : 11	5 : 3 : 15	—
WBL (weight-bearing line) ratio (%)	25.33 ± 5.66	24.49 ± 3.21	0.598
HKA (hip-knee-ankle angle) (°)	173.00 ± 1.95	173.83 ± 2.87	0.321
MPTA (medial proximal tibial angle) (°)	83.07 ± 1.52	83.50 ± 1.96	0.468
PTSA (posterior tibial slope angle) (°)	11.31 ± 1.91	10.04 ± 2.09	0.062
Insall–Salvati index	0.95 ± 0.11	1.01 ± 0.13	0.109

(PSI: patient-specific instrumentation. CO: conventional operation. BMI: body mass index).

**Table 2:** Comparison of imaging data.

	PSI group (*n* = 16)	CO group (*n* = 23)	*P* ^2^ value
WBL (weight-bearing line) ratio (%)			
(i) Preop target	60.16 ± 2.49	60.00 ± 3.11	0.868
(ii) Postop value	58.94 ± 3.35	56.67 ± 8.03	0.234
(iii) *P*^1^ value	0.063	0.017	—
(iv) Absolute difference from designed target	1.97 ± 1.83	5.42 ± 4.41	0.002
HKA (hip-knee-ankle angle) (°)			
(i) Preop target	183.56 ± 1.03	183.46 ± 1.12	0.765
(ii) Postop value	182.86 ± 1.20	181.73 ± 2.69	0.085
(iii) *P*^1^ value	0.038	0.003	—
(iv) Absolute difference from designed target	1.12 ± 0.86	2.27 ± 1.97	0.018
MPTA (medial proximal tibial angle) (°)			
(i) Preop target	91.33 ± 1.16	90.71 ± 0.65	0.072
(ii) Postop value	91.29 ± 1.49	90.45 ± 2.02	0.165
(iii) *P*^1^ value	0.917	0.503	—
(iv) Absolute difference from designed target	1.01 ± 0.57	1.48 ± 0.99	0.068
Correction angle (°)			
(i) Preop target	9.26 ± 2.09	9.33 ± 1.35	0.907
(ii) Postop value	8.96 ± 2.06	9.06 ± 1.32	0.868
(iii) *P*^1^ value	0.182	0.072	—
(iv) Absolute difference from designed target	0.66 ± 0.58	0.64 ± 0.38	0.912
PTSA (posterior tibial slope angle) (°)			
(i) Preop value	11.31 ± 1.90	10.05 ± 2.09	0.062
(ii) Postop value	11.33 ± 2.21	11.20 ± 2.49	0.866
(iii) *P*^1^ value	0.969	0.043	—
(iv) Absolute difference from preop values	1.30 ± 1.27	1.97 ± 1.99	0.244
Insall–Salvati index			
(i) Preop value	0.95 ± 0.11	0.97 ± 0.10	0.453
(ii) Postop value	0.96 ± 0.13	1.01 ± 0.13	0.271
(iii) *P*^1^ value	0.449	0.134	—

(PSI: patient-specific instrumentation. CO: conventional operation. Preop: preoperative. Postop: postoperative. *P*^1^, using paired *t* test. P^2^: using two-sample *t* test).

**Table 3:** Comparison of clinical outcomes.

		PSI group (*n* = 16)	CO group (*n* = 23)	*P* value
Operative duration (min)		109.38 ± 20.83	131.65 ± 29.92	0.014
Radiation exposures (n)		18.50 ± 4.80	28.22 ± 4.28	<0.001
Intraoperative blood loss (mL)		50.63 ± 14.36	97.82 ± 67.35	0.003
With and without BMP use/ Bone grafting (*n*)		10 : 6	11 : 12	0.516
Neural injury (*n*)		0	0	—
Lateral hinge fracture (*n*)		0	2	—
Infection (*n*)		0	0	—
Incisional exudation (*n*)		1	0	—
American Knee Society Score (AKSS)				
Clinical AKSS	Preop	57.50 ± 2.00	57.13 ± 2.39	0.616
	Postop (3 months)	75.63 ± 7.27	70.17 ± 8.37	0.042
	Postop (6 months)	85.00 ± 6.06	80.22 ± 7.42	0.040
	Last follow-up	90.31 ± 6.18	89.39 ± 6.33	0.654
Functional AKSS	Preop	58.75 ± 6.19	59.13 ± 7.63	0.870
	Postop (3 months)	70.93 ± 8.00	64.78 ± 8.98	0.034
	Postop (6 months)	81.56 ± 4.73	80.43 ± 8.25	0.625
	Last follow-up	90.00 ± 6.32	89.57 ± 6.38	0.835

(PSI: patient-specific instrumentation. CO: conventional operation. Preop: preoperative. Postop: postoperative).

## Data Availability

The data used to support the findings of this study are available from the corresponding author upon request.
